# No Increase in Acute or Late Radiation Toxicities in Patients With Ehlers-Danlos Syndrome Receiving Adjuvant Breast Radiation Therapy: A Report of Four Cases With Extended Follow-Up

**DOI:** 10.7759/cureus.50346

**Published:** 2023-12-11

**Authors:** Michael T Hsieh, Julan Amalaseelan, Abdul Rahim Mohd Tahir, Tue Le, Thomas P Shakespeare

**Affiliations:** 1 Radiation Oncology, Coffs Harbour Health Campus, Mid North Coast Local Health District, Coffs Harbour, AUS; 2 Radiation Oncology, North Coast Cancer Institute, Lismore, AUS

**Keywords:** radiation oncology complication, ehlers-danlos syndrome hypermobility type (eds-ht), intensity modulated radiation therapy (imrt), breast cancer radiation, radiotherapy toxicity, radiation therapy side effects, radiotherapy (rt), ehlers-danlos syndromes

## Abstract

Ehlers-Danlos syndrome (EDS) consists of a heterogeneous group of congenital collagen formation disorders characterised by skin hyperextensibility, atrophic scarring, and generalized joint hypermobility. Collagen vascular disorders have been implicated in increased incidence and severity of radiation toxicities; however, there are limited reports on the safety of radiation therapy with EDS. We identified all patients with EDS who received adjuvant conventional and hypofractionated breast radiation therapy at our institution and reviewed patient, treatment, and toxicity characteristics. Four patients were identified with a median follow-up of 13.2 months. Acute toxicities were limited to grade 1 dermatitis in all four patients. No late toxicities were seen. In this report, radiation therapy to the breast with conventional and hypofractionated regimens resulted in no significant acute or late toxicity.

## Introduction

Ehlers-Danlos syndrome (EDS) consists of a heterogeneous group of congenital collagen formation disorders with a prevalence of one in 2500 to one in 5000 patients, characterized by skin hyperextensibility, atrophic scarring, and generalized joint hypermobility [1,2]. The 2017 international classification of the EDSs describes 13 subtypes of EDS [1]. Collagen vascular disorders have been implicated in increased incidence and severity of radiation toxicities [3]. There have been few case reports on the safety of radiation therapy with EDS, and most of these have a very limited duration of follow-up and have used conventional fractionation [4-9]. Given potential concerns of increased radiation toxicity with EDS, we retrospectively examined the outcomes of patients with EDS who received adjuvant conventional or hypofractionated radiation therapy to the breast for breast cancer.

## Case presentation

Following ethics approval (NCNSW HREC 2019/ETH 12207) by the North Coast NSW Human Research Ethics Committee, electronic medical records (MOSAIQ, Elekta AB, Stockholm, Sweden) were searched from 2008-2023 to identify patients who were diagnosed with breast cancer, received adjuvant breast radiation therapy, and had a diagnosis of EDS. Baseline patient characteristics, treatment details, and treatment-related toxicity scores were prospectively scored by the treating radiation oncologist according to Common Terminology Criteria for Adverse Events version 5 (CTCAE v5).

In total, four eligible patients were identified. Baseline patient and treatment characteristics are detailed in Table 1. The median duration of follow-up was 13.2 months (range 1.2-62.9). The median age of patients was 56 years (range 46-71). One patient had a hypermobile subtype and three had a classic subtype of EDS. Surgically, three patients underwent lumpectomy and sentinel lymph node biopsy, followed by breast radiation therapy. One patient had a mastectomy and axillary lymph node dissection, followed by breast and supraclavicular fossa radiotherapy. All patients were treated with intensity-modulated radiation therapy. Two patients had left-sided breast cancers and two patients had right-sided breast cancers. Two patients received hypofractionated radiation therapy to a dose of 40Gy in 15 fractions over three weeks. Two patients received conventional fractionation to a dose of 50Gy in 25 fractions over five weeks. No patient received a tumour bed boost. Three patients were positioned supine, and one was positioned prone. One patient had partial breast radiotherapy. 

**Table 1:** Patient and treatment details ^*^according to the 2017 International Classification [1]; ^#^according to the American Joint Cancer Commission Staging, 8th Edition EDS: Ehlers-Danlos syndrome; IMRT: intensity-modulated radiation therapy

Patient characteristics	Patient 1	Patient 2	Patient 3	Patient 4
Age at diagnosis (years)	46	50	71	62
Type of EDS*	Hypermobile	Classic	Classic	Classic
Stage^#^	T1c N0 M0	T1c N1 M0	Tis N0 M0	T1c N0 M0
Surgery	Lumpectomy and sentinel lymph node biopsy	Lumpectomy and axillary dissection	Lumpectomy and sentinel lymph node biopsy	Lumpectomy and sentinel lymph node biopsy
Dose and fractionation (fx)	50Gy in 25 fx, no boost	50Gy in 25 fx, no boost	40Gy in 15 fx, no boost	40Gy in 15 fx, no boost
Target volume	Right whole breast	Right whole breast and supraclavicular fossa	Left whole breast	Left partial breast
Technique	IMRT	IMRT	IMRT	IMRT
Treatment position	Supine	Supine	Prone	Supine
Duration of follow-up (months)	62.9	22.3	4.3	1.2

The toxicity outcomes for our cohort are listed in Table 2. All patients were alive and cancer-free at the last follow-up. For acute toxicities, all four patients experienced grade 1 radiation dermatitis. No patient had grade 2 or above acute toxicities. All patients had excellent cosmesis at the time of the last follow-up. No late toxicities were reported.

**Table 2:** Acute and late toxicity grading according to CTCAE v5 CTCAE v5: Common Terminology Criteria for Adverse Events version 5

	Patient 1	Patient 2	Patient 3	Patient 4
Status at last follow-up	Alive, cancer-free	Alive, cancer-free	Alive, cancer-free	Alive, cancer-free
Acute toxicities				
Dermatitis	grade 1	grade 1	grade 1	grade 1
Pain	none	none	none	none
Late toxicities				
Poor cosmesis	none	none	none	none
Fibrosis	none	none	none	none
Breast lymphedema	none	none	none	none
Telangiectasia	none	none	none	none
Hypo- or hyper-pigmentation	none	none	none	none

## Discussion

EDS is a heterogeneous group of heritable connective tissue disorders, commonly characterized by gene mutations in COL5A1, COL5A2, COL1A1, and COL1A2, resulting in skin hyperextensibility, atrophic scarring, generalized joint hypermobility, easy bruising, skin fragility, and increased surgical complications, with a prevalence of one in 2500 to one in 5000 patients [1,2]. Various classification systems exist, including one from 1986, the Villefranche criteria in 1997, and the most recent 2017 international classification which lists 13 subtypes [1,10,11]. In particular, the vascular EDS subtype may impart increased morbidity, and one systematic review reported that 16-35% of vascular EDS patients present with cerebrovascular accidents, aneurysm and arterial rupture [12].

Collagen vascular disorders have also been implicated in increased incidence and severity of radiation toxicities [3]. However, there have been few case reports on the safety of radiation therapy with EDS, and most case reports have a very limited duration of follow-up and have used conventional fractionation [4-9]. Table 3 compares the current report with available MEDLINE (Medical Literature Analysis and Retrieval System Online)-indexed case reports of patients with EDS who have received radiation therapy. One concerning report from Holodny et al. noted severe grade 3 pericarditis, pleuritis, and mediastinitis during post-mastectomy radiation therapy, prompting treatment discontinuation at 10Gy [9]. Subsequently, this patient underwent whole-brain radiation therapy for brain metastases and reportedly died from a ruptured basilar artery aneurysm seven months after whole-brain radiation. An in vitro experiment by Aghajanyan et al. found increased radiation-induced chromosomal aberrations in irradiated lymphocytes of children with EDS when compared to that of control subjects, thus raising concerns about increased radiation damage to normal tissues [13]. However, apart from the aforementioned report by Holodny et al., all other case reports did not note any increased radiation toxicity and included radiation to a variety of sites including the axilla, supraclavicular fossa, mediastinum, breast, and brain [4-9]. 

**Table 3:** Comparison with published case reports ^*^treatment was discontinued at 10Gy due to severe acute toxicities

Study Author/Date	Ehlers-Danlos subtype	Number of patients	Age (years)	Radiation dose	RT technique	Site(s) treated	Follow-up (months)	Toxicity
Current report	Classic, Hypermobility,	4	Median 56 (46 – 71)	40-50Gy/15-25 fx, no boost	Intensity-modulated radiation therapy	Breast, supraclavicular fossa	Median 13.2 (1.2 – 62.9)	Acute: grade 1 dermatitis. Late: none
Chau and Chen, 2018 [5]	Hypermobility	1	42	46.8Gy/26 fx + 14Gy/7 fx boost	Photon Tangents + electron boost	Breast	0	Acute: grade 3 dermatitis. Late: not reported
Begbie et al., 1997 [4]	Vascular	1	50	50Gy/25 fx	Field-based	Anterior mediastinum	24	Acute: not reported. Late: not reported
Holodny et al., 1996 [9]	Hypermobile	1	62	Chest wall: 10Gy* Brain: 21Gy/14 fx	Field-based	Chest wall, Brain	7	Acute: pericarditis, pleuritis, mediastinitis. Late: death from ruptured basilar artery aneurysm 7 months post whole brain radiation
Deshpande et al., 2021 [6]	Hypermobile	1	62	Not reported	Protons	Axilla, supraclavicular fossa	Not reported	Acute: grade 2 dermatitis. Late: not reported
Falchook and Zagar, 2013 [7]	Hypermobile	1	71	20Gy/1 fx	CyberKnife	Brain – bilateral frontal lobe	6	Acute: none. Late: not reported
Sachinvala et al., 2018 [8]	Hypermobile	1	62	Unknown /30 fx	Fractionated stereotactic radiotherapy	Brain	Not reported	Acute: not reported. Late: not reported

To the best of our knowledge, the current report presents the highest number of case series to date, with the longest duration of follow-up, of women with EDS and breast cancer, treated with adjuvant conventional and hypofractionated radiation therapy to the breast. In this report of adjuvant breast irradiation, we did not detect any increased incidence or severity of acute or late radiation toxicities. Limitations of the study include the small number of patients, the heterogeneous nature of EDS, and the lack of genomic sequencing to subtype EDS in our patients. Future studies examining the safety of radiation according to each molecular subtype may be beneficial.

## Conclusions

To our knowledge, this is the largest reported series of patients with EDS undergoing adjuvant conventional and hypofractionated radiation therapy to the breast. No significant acute or late toxicities were seen in our cohort. Further research into the safety of radiation therapy for each subtype of EDS is warranted. Appropriate patient counselling regarding the safety of radiation therapy in a heterogeneous entity like EDS is recommended.
